# Dual Energy CT Angiography of Peripheral Arterial Disease: Feasibility of Using Lower Contrast Medium Volume

**DOI:** 10.1371/journal.pone.0139275

**Published:** 2015-09-29

**Authors:** Abdulrahman Almutairi, Zhonghua Sun, Abduljaleel Poovathumkadavi, Tarek Assar

**Affiliations:** 1 Department of Medical Radiation Sciences, School of Science, Curtin University, Perth, 6845, Western Australia, Australia; 2 Department of Medical Imaging, King Fahad Specialist Hospital, Dammam, 31444, Saudi Arabia; Shenzhen Institutes of Advanced Techology, CHINA

## Abstract

**Objective:**

One of the main drawbacks associated with Dual Energy Computed Tomography Angiography (DECTA) is the risk of developing contrast medium-induced nephropathy (CIN). The aim of the present study was firstly, to design an optimal CT imaging protocol by determining the feasibility of using a reduced contrast medium volume in peripheral arterial DECTA, and secondly, to compare the results with those obtained from using routine contrast medium volume.

**Methods:**

Thirty four patients underwent DECTA for the diagnosis of peripheral arterial disease. They were randomly divided into two groups: Group 1 (routine contrast volume group) with *n* = 17, injection rate 4–5 ml/s, and 1.5 ml/kg of contrast medium, and Group 2 ((low contrast volume group), with *n* = 17, injection rate 4–5ml/s, and contrast medium volume 0.75 ml/kg. A fast kilovoltage—switching 64-slice CT scanner in the dual-energy mode was employed for the study. A total of 6 datasets of monochromatic images at 50, 55, 60, 65, 70 and 75 keV levels were reconstructed with adaptive statistical iterative reconstruction (ASIR) at 50%. A 4-point scale was the tool for qualitative analysis of results. The two groups were compared and assessed quantitatively for image quality on the basis of signal-to-noise ratio (SNR) and contrast-to-noise-ratio (CNR). Radiation and contrast medium doses were also compared.

**Results:**

The overall mean CT attenuation and mean noise for all lower extremity body parts was significantly lower for the low volume contrast group (p<0.001), and varied significantly between groups (p = 0.001), body parts (p<0.001) and keVs (p<0.001). The interaction between group body parts was significant with CT attenuation and CNR (p = 0.002 and 0.003 respectively), and marginally significant with SNR (p = 0.047), with minimal changes noticed between the two groups. Group 2 (low contrast volume group) displayed the lowest image noise between 65 and 70 keV, recorded the highest SNR and CNR at 65 keV, and produced significantly lower results with respect to contrast medium volume and duration of contrast injection (p<0.001). The effect of radiation dose was not statistically significant between the two groups.

**Conclusions:**

DECTA images created at 65 keV and 50% ASIR with low contrast medium volume protocol, yielded results that were comparable to routine contrast medium volume, with acceptable diagnostic images produced during the evaluation of peripheral arteries.

## Introduction

Computed tomography angiography (CTA) has, over the last decade, become the preferred choice for diagnosing and evaluating peripheral arterial disease (PAD) in a manner comparable to invasive angiography [1]. Patients diagnosed with PAD are usually evaluated with CTA, which requires administration of iodinated contrast medium. The patient exposed to this technique faces the potential risk of developing contrast-induced nephropathy (CIN) [2–4]. With the onset of the 64-slice and post 64-slice CT era, scanning of the lower extremities could be achieved routinely in less than 10 seconds. However, the fast scan speed may outrun the contrast bolus, and therefore, large quantities of contrast volume are not essential for vascular studies [5]. Furthermore, low kilovoltage (kVp) levels have been shown to improve contrast enhancement in CTA [6, 7]. The disadvantages of this procedure lie in the beam hardening artefacts and the increase of image noise.

Dual-Energy CT (DECT) is the recently developed form of multidetector CT (MDCT) scanners, with the capability of compounding together two different tube voltages (kVp range 80–140). The main advantage of DECT is represented by material decomposition by acquiring two image series at the same anatomic location simultaneously with use of different kVp (80 and 140 kVp). Currently, there are three systems available for simultaneous acquisition of dual-energy images during a single breath-hold: 64-slice dual-source CT, 128-slice dual-source CT (Definition and Definition Flash, Siemens Medical Systems) and high-definition 64-MDCT (Discovery 750 HD, GE Healthcare) [8]. In the Siemens CT scanners, the two x-ray tubes use different kVp (80 and 140 kVp), while in the 64-MDCT, dual-energy imaging can be achieved with a single x-ray tube with fast kilovolt dynamic switching (from 80 to 140 kVp) between two different energy levels of x-rays from view to view during a single rotation. This improved technique overcomes the disadvantages of a single kVp, which is inherent in traditional MDCT scanners. The modification is brought about by combining the two high and low kVp voltages for generation of a variety of monochromatic CT images at multiple kilo-electron voltages (keV), ranging from 40 to 190 [9]. Consequently, the contrast volume is utilized most efficiently. Additional benefits of DECT include the facilitation of a number of post-processing opportunities such as the reduction of contrast material dose to the patients when low keV is applied [10, 11]. Appling the monochromatic images allows for the optimization of image quality parameters like image noise and CT attenuation, with low volume of contrast medium [11, 12].

Several reports have been published on the benefits of using (a) dual-source computed tomography (DSCT) that employs low contrast medium volume and /or concentration protocols for imaging different body regions including coronary artery, thoracic and abdominal aorta, and lower extremities, and (b) low-iodine concentration or low volume CTA showing good diagnostic images [13–21]. However, to the best of our knowledge, with respect to the diagnosis and assessment of PAD, the application of DECT and comparison of different amounts of both contrast medium volume and keV values, have not been fully reported. The present study aims at determining the optimal scanning protocol for DECT angiography (DECTA) in peripheral arterial imaging, by comparing two groups of patients who were separately administered routine and lower contrast medium volumes respectively, during DECTA scanning of the lower extremities. It was hypothesized that when weighed against the routine approach, lowering of the contrast medium volume would still produce acceptable diagnostic images, at the same time reducing the risk of CIN.

## Materials and Methods

### Patient population

Thirty-four patients (25 male and 9 female) between 27–73 years of age, with mean age, 52.73 ± 11.37 years, who had been advised to undergo peripheral arterial DECTA, were selected for the study. Duration of study period was September 2014 to March 2015. These subjects were randomly assigned to two groups as follows:

Group 1 or the routine contrast volume group, consisting of 17 patients in all (11 male and 6 female), with mean age 52.21 ± 13.55 years, falling within the age range of 27–73 years.

Group 2 or the low contrast volume group, consisting equally of 17 patients (14 male and 3 female), with mean age 53.35± 9.48 years, falling within the age range of 32–67 years.

Exclusion criteria for subjects included contraindication to intravenous administration of iodine contrast medium, and presence of renal dysfunction or renal failure. The study was approved by the Curtin Human Research Ethics (HR 167/2013) and King Fahad Specialist Hospital Committees (IRB-RAD029-FB). Informed written consent was obtained from all patients to participate in the study. The consent forms were kept in a secure location which was only accessed by investigators. The Institutional Review Boards (IRBs) approved this consent procedure.

### CT scanning protocol

All CT examinations were performed on a fast kilovoltage-switching 64-slice CT scanner (Discovery CT HD 750; Gemstone Spectral Imaging, GE Healthcare, Milwaukee, Wisconsin, USA) in the DE mode. The following features of GSI (Gemstone Spectral Imaging)-48 protocol were used:

- collimation—64 × 0.625 mm,
- pitch—0.984,
- gantry rotation time—0.7s,
- slice thickness—1 mm,
- reconstruction interval—1 mm,
- alternated 80 kVp and 140 kVp with the same X-ray tube by fast kVp switching,
- a constant tube current of 600 mAs, (tube current modulation is not available in DE acquisition in this system).

A total of 6 sets of monochromatic images at 50, 55, 60, 65, 70 and 75 keV were reconstructed with adaptive statistical iterative reconstruction (ASIR) at 50%.

The non-ionic intravenous contrast medium namely, Xenetix 350® (350 mg. Iodine/mL, Guerbet, Sulzbach, Germany), was injected using a power injector (Envision CT injector, Medrad) through an 18–20G catheter inserted into the median cubital vein. A bolus tracking technique was used to initiate the scan in the abdominal aorta at the level of the celiac trunk with a threshold of 150 HU. Group 1 (routine group) and Group 2 (low contrast group) were respectively administered 1.5 mL/kg bogy weight and 0.75mL/kg body weight (a reduction of about 50%) of the mentioned contrast medium, followed by 40 ml of a saline flush. The injection rate of contrast medium and saline solution was 4–5 ml/s for all subjects in both groups

### Qualitative assessment of image quality

Two radiologists each with 20 years and 15 years of experience in body imaging and CTA interpretation, performed qualitative evaluations separately on a workstation with dedicated software (Gemstone Spectral Imaging Viewer, GE). The different virtual monochromatic spectral (VMS) image sets of each individual patient were evaluated randomly at 50, 55, 60, 65, 70 and 75 keV energy levels by each reader. It was deemed unnecessary to blind the readers to the altered energy levels because the different values could easily be detected by visual inspection of the images.

The image quality of the different VMS series, as well as the 2D and 3D reformations (Maximum-Intensity Projection (MIP) and Multiplanar Reformations (MPR)), were subjectively analyzed using a 4-point scale, where the scores were interpreted as follows: 1—poor vessel opacification and non-diagnostic; 2—fair vessel opacification; 3—good vessel opacification, and 4—excellent vessel opacification. The evaluation was carried out for three body regions, namely, the pelvic, thigh and leg regions. A score of 2 or above 2 was considered clinically diagnostic.

### Quantitative assessment of image quality

A single reviewer performed quantitative analysis on the same workstation. The CT attenuation (mean CT number in Hounsfield units) and noise (computed as standard deviation of the CT number in Hounsfield units) were calculated for the main peripheral arteries including: the common iliac, superficial femoral and tibial arteries, by placing a defined Region of Interest (ROI) on all VMS series. ROIs were marked as large as possible in the vessel lumen. Areas with wall calcification that might cause artifacts, were avoided. The CT attenuation of the background was also measured for all patients at the region of the muscle closest to the arteries under investigation. The mean CT attenuation and noise were calculated for individual subjects by averaging the values derived from both sides of the arteries under study. The Signal-to-Noise Ratio (SNR) was calculated as mean CT value of ROI divided by the mean image noise (SD), while the Contrast-to-Noise Ratio (CNR) was calculated as mean CT value of vessel minus CT value of background muscle divided by the mean image noise (SD) of vessel, which is CNR = (Mean _vessel_−Mean _muscle_)/SD_vessel_.

### Radiation dose estimation

To arrive at the most effective radiation dose, the scan length was documented for each patient. Thereafter, the volume CT dose index (CTD vol) and dose length product (DLP) were recorded from the CT console following individual examination of subjects. The multiplication product of DLP and a conversion factor for peripheral arteries examinations in the lower extremities [k = 0.0056 mSv/ (mGy × cm)] yielded the effective dose of radiation [22].

### Statistical analysis

Statistical analysis was performed with the help of commercial software SPSS version 22.0, (SPSS Inc., Chicago, Illinois, USA). Quantitative variables were expressed as the mean ± SD and categorical variables such as frequencies or percentages. Repeated measures (split plot) Analysis of Variance (ANOVA) was used to compare the CT value, noise, SNR and CNR of the VMS images at varying monochromatic energy levels. The analysis of covariance (ANCOVA) was used to compare six factors (group, gender, hypertension, smoking, Diabetes, and hyperlipidemia), with dependent variables such as scan time, scan range, DLP and effective dose. At the main plot level, the design was simple and fully randomized. Subsequently, each participant was ‘split’ into 18 sub-plots, corresponding to the 18 combinations of 3 body regions with 6 voltage settings, to enable the applying of, 18 regimes to each of the participants and facilitate recording of output measure again, for each regime (hence the name, repeated measures). This experimental design produced a total of 34 x 18 = 612 sub plots. A high degree of calcification in the arteries of the thigh and leg regions of two patients belonging to Group 2 (low contrast medium group) made it necessary to exclude them from the analysis of quantitative image quality. A measure of the concurrence between the two radiologists for various parameters was elicited with kappa coefficient of concordance, which provided information about inter-observer variability. Probability values of less than 0.05 were considered statistically significant.

## Results

Scans were successfully completed for each of the 34 enrolled patients. Table 1 displays patient characteristics and contrast protocols.

**Table 1:** Patient characteristics and contrast protocols.

	Routine contrast volume (n = 17)	Low contrast volume (n = 17)
**Patient characteristics**		
Male: Female	11:6	14:3
Age, yr	52.12 ± 13.17	53.35 ± 9.21
Weight, (kg)	77.07 ± 15.56	71.49 ± 13.87
Height, (cm)	162.65 ± 7.78	163.68 ± 8.55
BMI, (kg/m^2^)	29.84 ± 5.77	26.79 ± 5.49
**Contrast medium**		
Contrast volume, (mL)	116.00 ± 16.09	66.47 ± 6.83***
Flow rate, (mL/sec)	4.74 ± 0.35	4.76 ± 0.39
Contrast duration, (sec)	25.01 ± 3.37	14.69 ± 2.34***
**Scanning parameters and radiation dose**		
Scanning time, (sec)	24.27 ± 1.52	24.64 ± 1.16
Scanning range, (mm)	1244.18 ± 103.03	1272.12 ± 67.09
DLP (mGy^.^cm)	1238.52 ± 73.25	1257.53 ± 58.45
Effective dose (mSv)	7.56 ± 0.53	7.57 ± 0.80

Conversion factor for peripheral CTA = 0.0056 mSv/mGy*cm

BMI, body mass index; DLP, dose length product

*** p<0.001, highly significant

### Scan time

Analysis of risk factor demonstrated that there were four factors affecting scan time: (a) *Hypertension*—a significant effect (p = 0.013) was seen to be exerted by hypertension, with the scanning time increasing from 23.5 seconds (non-hypertensives) to 24.5 seconds (hypertensives); (b) *Diabetes*—a highly significant effect (p = 0.004) was observed in the presence of Diabetes (diabetics vs non-diabetics 24.9 secs vs 23.4 secs); (c) *Age*—every additional year of age was reflected in a reduction of scanning time by 0.038 seconds. The statistical test of significance was positive with p value being 0.032; (d) *Body weight*—a highly significant difference (p<0.001), was noted with each additional kilogram of body weight with an increase of 0.47 seconds (S1, S2 and S3 Tables).

### Image quality assessment

Assessment of image quality was carried out by taking the overall mean CT value and mean noise for all body regions. It was seen to be significantly lower for low volume contrast group than for the routine contrast group (p <0.001). A more detailed account of this parameter follows.

In both groups, the CT value in the pelvis, thighs, and legs for all the keV ranges was >230HU, which is considered sufficient opacification for CTA examination. *CT attenuation* varied significantly within groups (p = 0.001), body parts (p<0.001) and keVs (p<0.001). The interaction between group and Body part in terms of CT attenuation and CNR was significant (p = 0.002 and p = 0.003 respectively, Table 2). However, the mean CT attenuation was lower for the low contrast volume group than for the routine contrast volume group, recording statistically significant figures (p = 0.001). The marked difference in interaction between body part and the two contrast groups was reflected in a huge drop from 494 HU (routine/pelvis) to 361 HU (low/pelvis). An even larger drop was observed for the thigh region, and the largest drop was noticed for legs. These results were similar for both the groups. The SNR in contrast, remained slightly different, with a marginally significant p value (0.047) (Tables 3 and 4).

**Table 2:** Results of factorial ANOVA.

		CT value		Image noise		SNR		CNR	
Source		F	p-value	F	p-value	F	p-value	F	p-value
Group	Hypothesis	**67.898**	**0.001**	**19.115**	**0.001**	**15.897**	**0.001**	**20.976**	**0.001**
Body Part	Hypothesis	**18.240**	**0.001**	**45.943**	**0.001**	**42.011**	**0.001**	**172.846**	**0.001**
KeV	Hypothesis	**597.903**	**0.001**	**97.788**	**0.001**	**4.863**	**0.001**	**19.767**	**0.001**
Group * Body Part	Hypothesis	**6.352**	**0.002**	**2.684**	**0.072**	**3.071**	**0.047**	**5.904**	**0.003**
Body Part * keV	Hypothesis	**1.021**	**0.424**	**1.066**	**0.387**	**0.671**	**0.751**	**0.660**	**0.762**
Group * keV	Hypothesis	**17.543**	**0.001**	**0.863**	**0.506**	**0.169**	**0.974**	**0.092**	**0.993**
Group * Body Part * keV	Hypothesis	**0.362**	**0.962**	**0.039**	**1.000**	**0.077**	**1.000**	**0.041**	**1.000**

Group: routine contrast volume and low contrast volume, CT value: CT number in Hounsfield units, SNR: Signal-to-noise ratio in the stented area, CNR: Contrast-to-noise ratio, F: value of test statistic of F-test for corresponding effect; *P*: corresponding p-value, keV: kiloelectron volt.

**Table 3:** Quantitative and qualitative assessment of image quality at different body parts using routine and low contrast volumes.

Body Part/ Contrast groups	keV	CT value	Image noise	SNR	CNR	Likert score
1 Pelvis/Routine contrast volume	**50**	**737.29 ± 138.88**	**62.56 ± 19.77**	**12.59 ± 3.44**	**13.37 ± 4.80**	**3.88 ± 0.33**
1 Pelvis/Routine contrast volume	**55**	**614.29 ± 115.27**	**53.05 ± 16.72**	**12.36 ± 3.36**	**12.91 ± 4.51**	**3.32 ± 0.67**
1 Pelvis/Routine contrast volume	**60**	**510.37 ± 94.07**	**37.44 ± 11.37**	**14.51 ± 3.68**	**15.90 ± 5.55**	**3.32 ± 0.72**
1 Pelvis/Routine contrast volume	**65**	**426.08 ± 77.49**	**30.17 ± 8.31**	**14.89 ± 3.69**	**15.36 ± 4.79**	**3.20 ± 0.82**
1 Pelvis/Routine contrast volume	**70**	**363.98 ± 64.53**	**27.70 ± 7.56**	**13.82 ± 3.43**	**13.89 ± 4.46**	**2.88 ± 1.10**
1 Pelvis/Routine contrast volume	**75**	**316.16 ± 57.68**	**29.59 ± 7.91**	**11.20 ± 2.92**	**10.49 ± 3.65**	**2.82 ± 1.11**
1 Pelvis/Low contrast volume	**50**	**536.26 ± 157.29**	**55.13 ± 17.78**	**10.20 ± 3.22**	**10.60 ± 4.10**	**3.64 ± 0.55**
1 Pelvis/Low contrast volume	**55**	**447.18 ± 130.39**	**47.09 ± 14.96**	**9.98 ± 3.23**	**10.07 ± 3.73**	**2.73 ± 0.96**
1 Pelvis/Low contrast volume	**60**	**373.45 ± 106.48**	**32.22 ± 11.66**	**12.37 ± 4.17**	**12.32 ± 5.60**	**3.14 ± 0.95**
1 Pelvis/Low contrast volume	**65**	**312.60 ± 87.29**	**25.61 ± 9.28**	**13.18 ± 4.73**	**12.48 ± 6.59**	**2.82 ± 0.97**
1 Pelvis/Low contrast volume	**70**	**267.08 ± 74.83**	**24.17 ± 7.83**	**11.85 ± 4.33**	**10.28 ± 4.41**	**2.53 ± 1.02**
1 Pelvis/Low contrast volume	**75**	**230.74 ± 64.61**	**26.17 ± 7.68**	**9.58 ± 4.11**	**7.73 ± 3.15**	**1.91 ± 0.97**
2 Thigh/Routine contrast volume	**50**	**745.29 ± 184.23**	**56.04 ± 28.81**	**15.42 ± 6.30**	**24.07 ± 11.70**	**3.61 ± 0.58**
2 Thigh/Routine contrast volume	**55**	**622.78 ± 150.15**	**47.19 ± 24.28**	**15.26 ± 6.05**	**23.24 ± 11.05**	**3.05 ± 0.80**
2 Thigh/Routine contrast volume	**60**	**517.80 ± 122.39**	**36.52 ± 19.57**	**16.67 ± 6.64**	**28.04 ± 12.67**	**3.44 ± 0.70**
2 Thigh/Routine contrast volume	**65**	**432.90 ± 101.88**	**30.16 ± 15.95**	**17.09 ± 7.39**	**28.96 ± 12.97**	**2.99 ± 0.90**
2 Thigh/Routine contrast volume	**70**	**368.04 ± 97.45**	**26.18 ± 14.29**	**16.75 ± 6.94**	**25.98 ± 11.84**	**2.85 ± 1.00**
2 Thigh/Routine contrast volume	**75**	**328.29 ± 74.25**	**25.01 ± 12.75**	**15.11 ± 5.55**	**19.88 ± 9.39**	**3.02 ± 1.18**
2 Thigh/Low contrast volume	**50**	**508.48 ± 178.71**	**40.04 ± 12.06**	**13.57 ± 5.57**	**16.77 ± 7.49**	**3.41 ± 0.79**
2 Thigh/Low contrast volume	**55**	**430.54 ± 146.11**	**33.68 ± 10.31**	**13.70 ± 5.46**	**16.30 ± 6.96**	**2.38 ± 0.93**
2 Thigh/Low contrast volume	**60**	**360.21 ± 121.88**	**25.05 ± 9.36**	**16.08 ± 7.23**	**20.98 ± 9.73**	**3.11 ± 1.05**
2 Thigh/Low contrast volume	**65**	**304.78 ± 102.39**	**20.65 ± 9.24**	**17.36 ± 8.38**	**22.43 ± 11.20**	**2.47 ± 0.99**
2 Thigh/Low contrast volume	**70**	**266.48 ± 86.83**	**17.87 ± 6.63**	**16.55 ± 6.93**	**17.99 ± 8.21**	**2.67 ± 1.07**
2 Thigh/Low contrast volume	**75**	**235.53 ± 74.43**	**17.55 ± 5.48**	**14.49 ± 5.65**	**13.34 ± 5.54**	**2.05 ± 0.97**
3 Legs/Routine contrast volume	**50**	**665.62 ± 185.87**	**72.80 ± 31.41**	**11.27 ± 6.72**	**24.43 ± 11.81**	**3.29 ± 0.94**
3 Legs/Routine contrast volume	**55**	**566.85 ± 155.02**	**61.67 ± 26.63**	**11.32 ± 6.66**	**23.64 ± 11.39**	**3.49 ± 0.74**
3 Legs/Routine contrast volume	**60**	**460.82 ± 131.12**	**48.06 ± 20.94**	**11.99 ± 7.38**	**28.33 ± 13.76**	**3.29 ± 0.92**
3 Legs/Routine contrast volume	**65**	**385.55 ± 114.27**	**38.75 ± 16.99**	**12.51 ± 7.83**	**28.96 ± 13.81**	**3.11 ± 0.78**
3 Legs/Routine contrast volume	**70**	**342.71 ± 97.26**	**35.08 ± 15.53**	**12.29 ± 7.49**	**24.72 ± 12.64**	**2.82 ± 0.84**
3 Legs/Routine contrast volume	**75**	**311.92 ± 86.02**	**32.43 ± 14.33**	**11.94 ± 6.81**	**20.27 ± 10.44**	**2.35± 1.02**
3 Legs/Low contrastvolume	**50**	**500.62 ± 147.36**	**61.14 ± 43.33**	**11.39 ± 8.40**	**18.63 ± 7.31**	**3.50 ± 089**
3 Legs/Low contrast volume	**55**	**425.86 ± 124.15**	**51.68 ± 36.71**	**11.62 ± 8.55**	**18.11 ± 7.03**	**2.64 ± 0.88**
3 Legs/Low contrast volume	**60**	**348.15 ± 99.60**	**39.74 ± 28.57**	**11.99 ± 7.21**	**21.83 ± 8.43**	**2.96 ± 1.15**
3 Legs/Low contrast volume	**65**	**289.41 ± 82.79**	**32.00 ± 23.72**	**12.60 ± 7.17**	**22.29 ± 8.74**	**2.65 ± 0.82**
3 Legs/Low contrast volume	**70**	**257.90 ± 74.52**	**29.25 ± 21.98**	**12.62 ± 7.71**	**18.84 ± 7.56**	**2.29 ± 1.12**
3 Legs/Low contrast volume	**75**	**232.62 ± 68.02**	**27.55 ± 23.72**	**12.38 ± 8.18**	**15.01 ± 6.23**	**2.11 ± 0.98**

**Table 4:** CT value, Image noise, SNR and CNR.

Body part	Contrast groups	CT value	Image noise	SNR	CNR
1 Pelvis/Routine contrast volume		**494.69** ^a^	**40.07** ^a^	**13.23** ^a^	**13.66** ^a^
1 Pelvis/Low contrast volume		**361.22** ^a^ **(428)** ^b^	**35.07** ^a^ **(37.6)** ^b^	**11.19** ^a^ **(12.2)** ^b^	**10.58** ^a^ **(12.1)** ^b^
2 Thigh/Routine contrast volume		**502.52** ^a^	**36.85** ^a^	**16.05** ^a^	**25.03** ^a^
2 Thigh/Low contrast volume		**351.00** ^a^ **(427)** ^b^	**25.81** ^a^ **(31.3)** ^b^	**15.29** ^a^ **(15.7)** ^b^	**17.97** ^a^ **(21.5)** ^b^
3 Legs/Routine contrast volume		**455.58** ^a^	**48.13** ^a^	**11.89** ^a^	**25.06** ^a^
3 Legs/Low contrast volume		**342.43** ^a^ **(399)** ^b^	**40.23** ^a^ **(44.2)** ^b^	**12.10** ^a^ **(12)** ^b^	**19.12** ^a^ **(22.1)** ^b^
Total/Routine contrast volume		**484.30** ^a^	**41.70** ^a^	**13.70** ^a^	**21.30** ^a^
Total/Low contrast volume		**351.60** ^a^	**33.70** ^a^	**12.90** ^a^	**15.90** ^a^

a) The body parts mean values.

b) Based on modified population marginal means after excluding the 2 cases with high calcification in the thigh and leg regions

No evidence of interaction effects was observed between body part and keV, nor between groups and body part by keV for the entire range of quality variables. The interaction between group by keV was highly significant (p<0.001). Reduction in CT values between routine and low contrast groups, with increase in voltage were 28.1%, 27.8%, 27.3%, 27.1%, 26.3% and 26.9% HU respectively, so it can be inferred that the difference between routine and low contrast uniformly came down with a rise in voltage.

**Fig 1:**
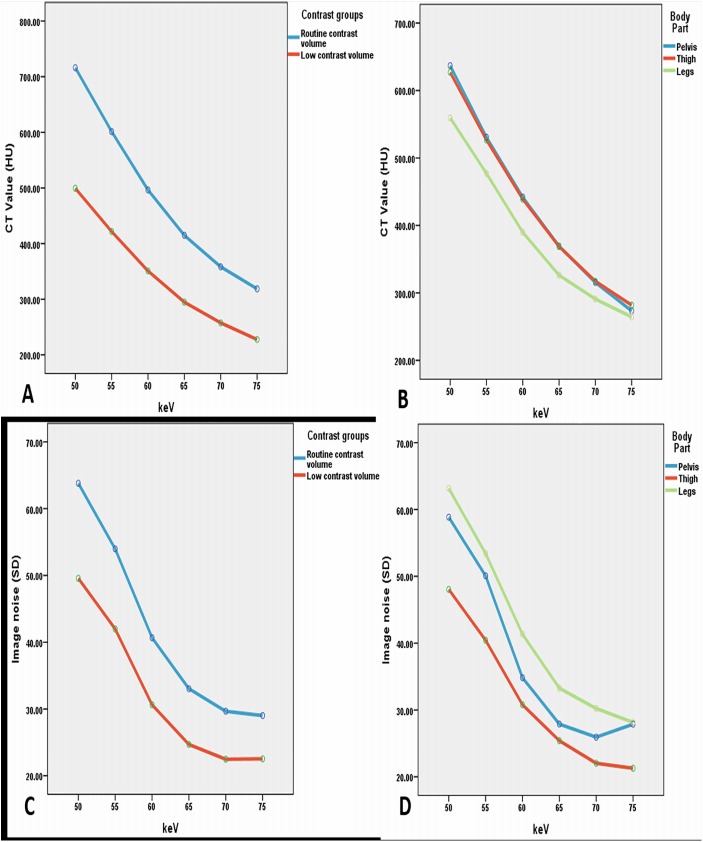
Comparison of CT attenuation and image noise measured at two contrast groups at different body parts with variable keV sets. A: Comparison of the measured CT values in the monochromatic images for the two contrast groups. B: comparison of the measured CT values of three body parts at different keV sets. C: image noise values in the monochromatic images for the two contrast groups. D: comparison of image noise of different body parts at different keV sets.

**Fig 2:**
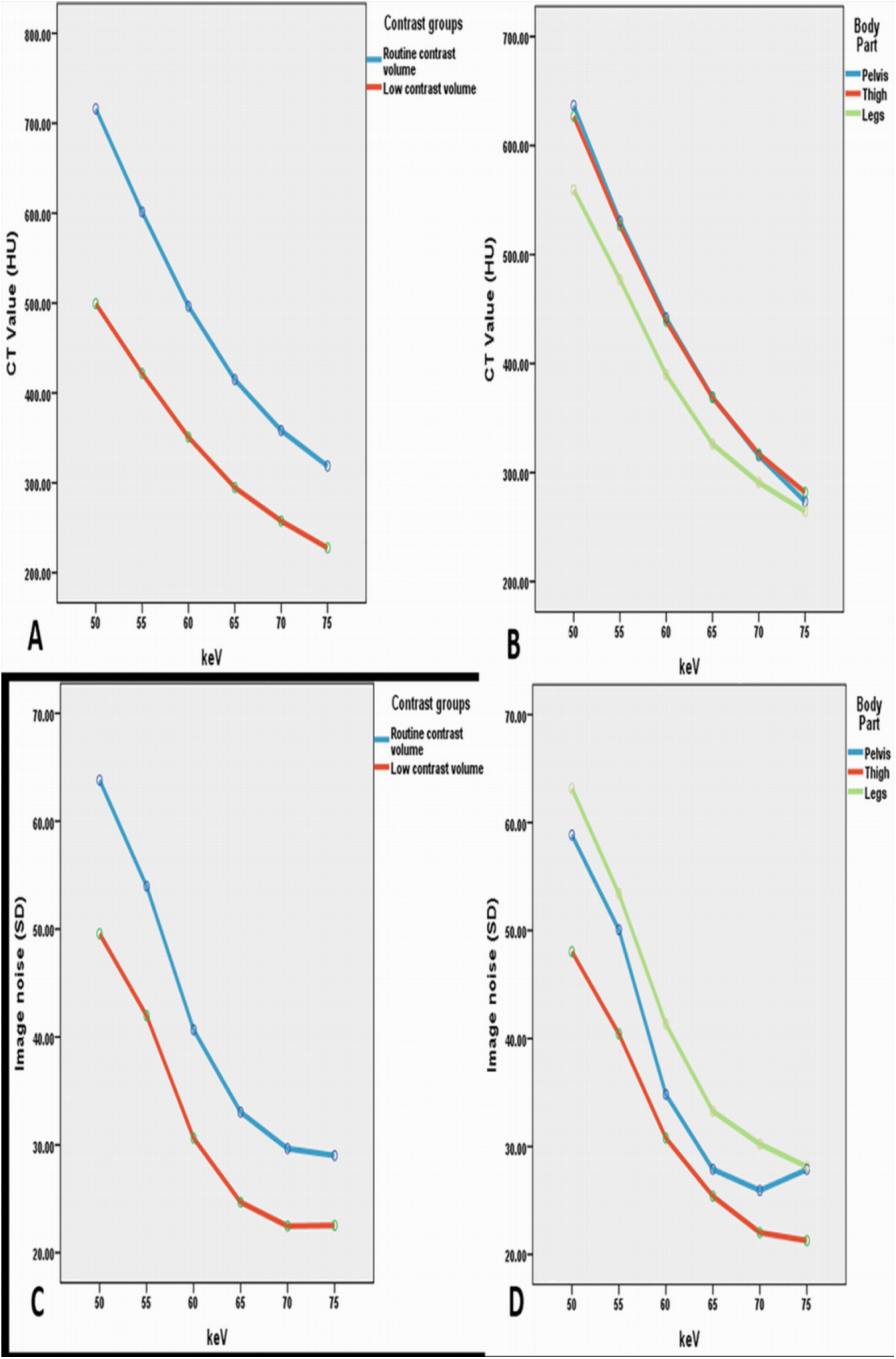
Comparison of SNR and CNR measured at two contrast groups at different body parts with variable keV sets. Comparison of calculated and measured SNR in monochromatic images with A showing the differences between the two contrast groups at different keV sets, B representing the SNR values of keV sets with three body parts, C showing the comparison of calculated and measured CNR in monochromatic images for the two contrast groups, and D demonstrating the CNR values of three body parts at different keV sets. In the range of 55–65 keV, both of the two curves increase sharply with the gradual rise in keV. Between 65 and 75 keV, both curves of the contrast values decrease sharply with 65 keV resulting in the highest value. SNR: signal-to-noise ratio, CNR: contrast-to-noise ratio.

**Fig 3:**
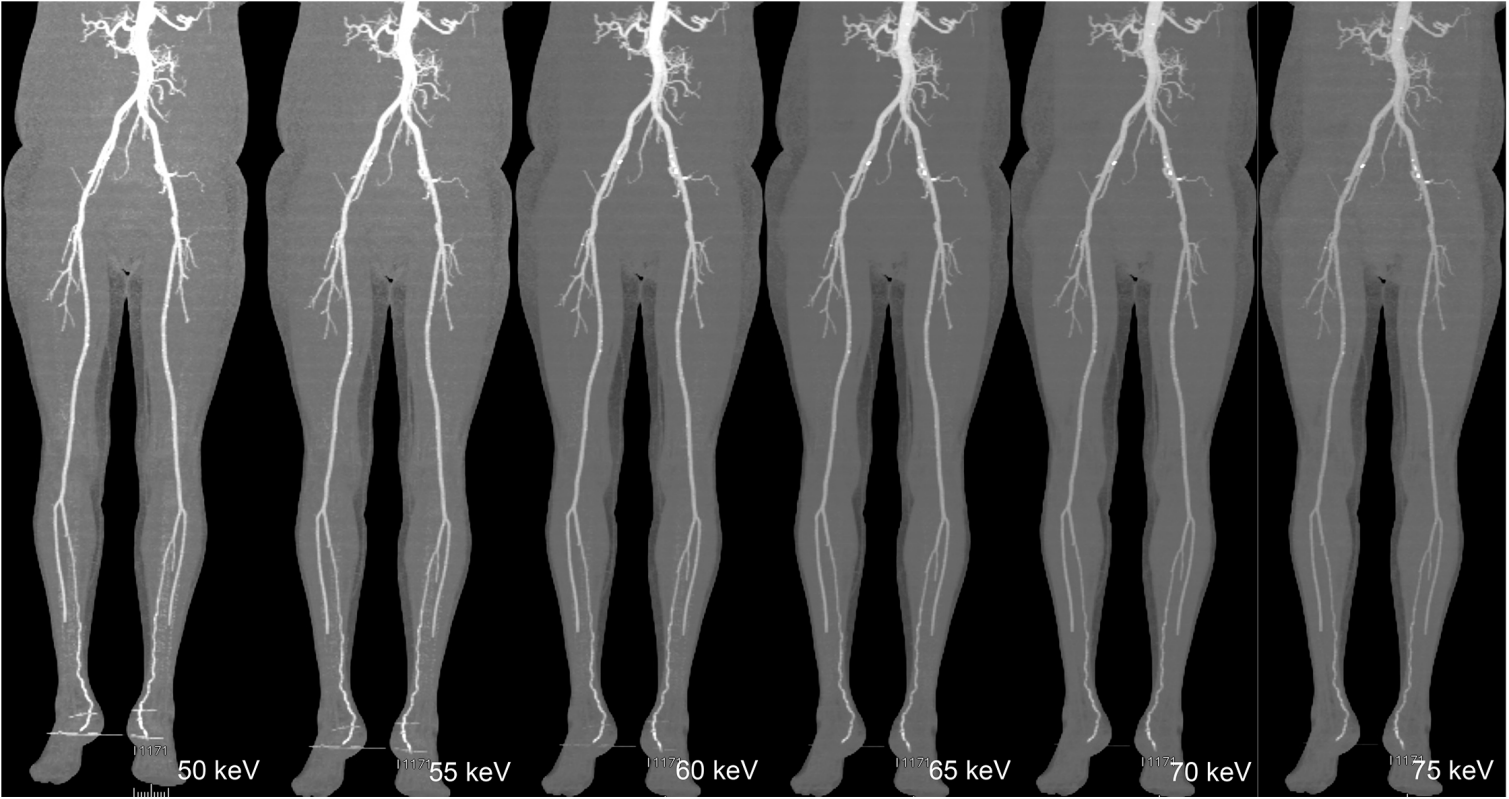
DECTA images acquired with different keV values using routine contrast medium. Examples of image quality of DECTA maximum-intensity projection (MIP) were shown in a 53-year-old female with body weight of 54 kg using 80 ml of contrast medium, Comparison among DECTA acquisitions in the different virtual monochromatic energies (50, 55, 60, 65, 70 and 75 keV) shows higher image noise at 50 and 55 keV which affects visualization of the vascular lumen details. VMS images acquired at 65 keV were shown to have better image quality (higher SNR and CNR) compared to the other keVs. DECTA: dual-energy CT angiography, VMS: virtual monochromatic spectral.

**Fig 4:**
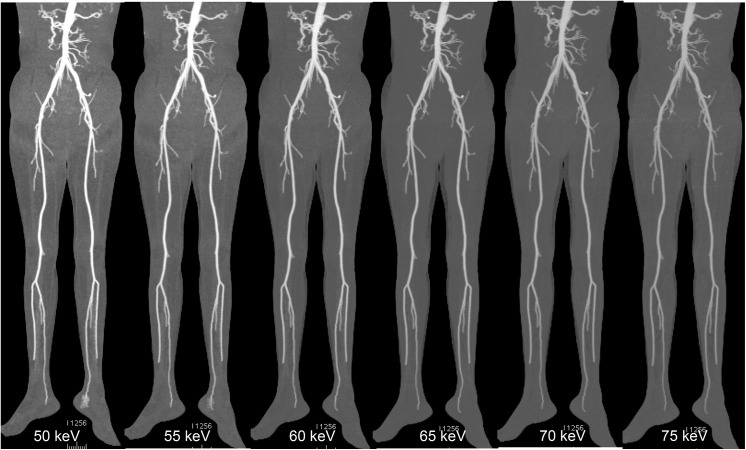
DECTA images acquired with different keV values using low contrast medium. A series of MIP images of DECTA were obtained in a 43-year-old male with body weight of 56 kg using 50 ml of contrast medium in the diagnostic assessment of peripheral arteries. Comparison among DECTA acquisitions in the different virtual monochromatic energies (50, 55, 60, 65, 70 and 75 keV) shows higher image noise at 50 and 55 keV which affects visualization of the vascular lumen details. VMS images acquired at 65 keV were shown to have better image quality (higher SNR and CNR) compared to the other keVs. DECTA: dual-energy CT angiography, MIP-maximum-intensity projection, VMS: virtual monochromatic spectral.

Image noise was found to be less with low contrast volume group at 65 to 70 keV, but when it reached the 70 keV energy level, the noise remained either unchanged or increased to a small extent in the pelvic area (Fig 1). The highest values of SNR and CNR were obtained at 65 keV for both groups. Fig 1 portrays an example of image quality with different keV ranges, and Fig 2 displays the comparison of SNR and CNR figures across the keV ranges. Figs 3 and 4 show examples of DECTA images acquired with different keV ranges using routine and low contrast medium.

With respect to qualitative analysis, the mean score of the CTA image quality indices for routine contrast volume and low contrast volume groups were 3.24 and 2.80, respectively. The lowest quality in both groups was attained in the leg region due to the incidence of severe calcification. Inter-reader concurrence for image quality grading was moderate (k = 0.33). Overall, the qualitative assessment of image quality indicated that all the images were acceptable for clinical diagnosis.

### Radiation dose

The difference in radiation dose was not statistically significant (DLP and effective dose) between the routine contrast group and low contrast group cohort (p = 0.07). Routine contrast group figures were 1238.52 ± 73.25 mGy*cm and 7.56 ± 0.53 mSv (male: 7.36 ± 0.53 mSv, female: 7.93 ± 0.24 mSv), while the low contrast group readings were 1257.53 ± 58.45 mGy*cm and 7.57 ± 0.67 mSv (male: 7.46 ± 0.78 mSv, female: 8.12 ± 0.68 mSv).

## Discussion

The present study compared low volume with routine volume contrast medium for assessment of peripheral arteries using DECT on a clinical patient population, with the aim of evaluating the optimal contrast protocol of image quality, using virtual manipulation of keV-settings. To the best of our knowledge, no studies have been published comparing different contrast volumes with varying keV settings in DECTA of lower extremities. Results of the investigation demonstrated that contrast medium volume could be reduced by as much as 50% without compromising on vascular visualization. Furthermore, a DECTA protocol of 65-keV with 50% ASIR resulted in the highest degree of CT attenuation and lowest image noise, with resultant increase in SNR and CNR, in comparison with the other VMS levels attained in both the groups.

The findings of the study were in consistency with previous reports in a phantom study, which illustrated the achievement of optimal image quality between 65 and 70 keV [23]. From the results of the present experiment, it was obvious that lower keV in peripheral DECTA produced higher vascular enhancement in both groups, irrespective of contrast volume. The investigation reiterates the feasibility of reducing contrast medium volume by 50% without negatively altering diagnostic image quality. These findings are supported by Baxa et al., [17], who used only 40mL in their study of lower extremity DECTA imaging. The researchers however, made no attempt to evaluate spectral imaging because they assessed contrast delivery rather than scanning techniques. In other words, reduction of contrast medium volume was not a priority, and therefore, there was no significant difference in medium volume between control (routine) and experimental (low dose) groups. Thus, the image quality at 65 keV, for both groups, was found to be optimal for lower extremity CTA with Dual Energy CT. This protocol could greatly minimize the risk of CIN in patients with renal insufficiency.

In recent years, a number of studies have focused on image quality using different virtual keV-values in imaging body vasculature [9, 24, 25]. Of them only one study pertained to the evaluation of peripheral arteries through DECT at varying keV levels [9]. Yet, authors discovered that best image quality for lower extremity CTA was attainable at 60 keV. Another study on abdominal CTA imaging with altered contrast volumes showed that up to 70% of contrast volume could be used successfully with DECT. Some other research workers have reported that contrast medium volume determined the optimal keV level [25]. Contrary to these findings, the present investigation demonstrated that an inferior image quality was produced at 60 keV compared to 65 keV. This observation is supported by the postulation that 60 keV is the outcome of the 80 kVp, which corresponds to higher image noise [26].

Several researchers have worked on the efficacy of VMS for visualizing thoracic and abdominal arteries. Delesalle et al., studied spectral optimization of thoracic arteries and found that 60 keV and 100 keV produced either identical or better image quality when compared to standard chest CTA [18]. Maturen et al., in their investigation on endovascular aneurysm of the aorta, established high sensitivity in the detection of endoleaks at 55 keV when compared to standard CTA [27]. In another study, Sudarski et al., recommended that using 70 keV may achieve a higher CNR in abdominal arteries [9]. Pehno et al., compared the image quality of VMS DECTA with standard CTA in aortoillic arteries, and demonstrated that optimal contrast enhancement improved image quality at 70 keV [24]. Applying these values for lower extremity DECTA may however, not be feasible because of inherent structural differences in body regions. The present undertaking used 65 keV to attain a lower image noise and higher CNR and SNR, in order to promote efficient imaging of peripheral arteries.

The type and severity of vascular disease has enormous impact on blood flow velocity and contrast delivery. Improved image quality has been achieved in patients with PAD, by employing faster table speed. In contrast, a lower table speed gives rise to better image quality in patients with abdominal aortic aneurysm [28]. It has been reported that aneurismal patients exhibit longer aortic peaks compared to non-aneurismal subjects [17]. With regard to duration of scan period, it was found in the present investigation that hypertensive patients needed a lower scan time compared to non-hypertensives, and conversely, non-diabetic patients required a significantly shorter scan time than diabetic patients. Age and body weight were seen to be important influencing factors, producing significantly different readings for scan time. Clinicians need to consider all these findings while scanning patients with different risk factors so that the best possible desired CTA images may be obtained. On a cautionary note, it must be mentioned that more in-depth studies are recommended in this area due to the limited number of respondents participating in the present study.

Radiation dose did not show any marked difference between the experimental (lower dose) and control (routine dose) groups, as a result of differences in DLP. The effective dose in this study was about 7.6 mSv for both groups, which is slightly higher than the 5.74 mSv as reported by Dong et al [21], but it is similar to the 7.0 mSv for DECT pulmonary angiography in a prospective randomized trial [4]. According to the effective dose range of various CT procedures [29], the radiation dose of DECTA in this study is within the acceptable limit, although further dose reduction could be achieved with the advancement of new generation DECT. Furthermore, the effective dose in this study was calculated according to the latest methodology, and appeared to be smaller than quantities used in previous studies because of the application of a new conversion coefficient for DECT of the lower extremities. Consequently, interpretation of results from this report needs to be carried out with care, particularly when making comparisons with previously reported results in literature.

### Limitations of the study

The present study is not without limitations:

- A single scanning center was approached and a relatively small number of patients were incorporated into the study. The reason for this is the still emerging role of DECTA in PAD, which rendered recruitment of more number of patients difficult and challenging. This limitation could be overcome with further multi-center studies.
- No comparison has been made of DECTA with invasive angiography, which is regarded as the reference method for arterial imaging. As a result, no assurance of diagnostic accuracy is available in this study. The potential risk of radiation exposure and the use of high volume contrast medium during invasive angiography, are responsible for its gradual replacement by less invasive and safer modalities such as CTA.
- Measurement of attenuation was performed at only three levels. This may be justified by the fact that the inclusion of numerous variables led to the evolvement of a combination of more than 600 subplots, thereby making the results suitable for qualitative and quantitative analysis.
- DECT application being at the developing stage in routine clinical practice, readers are still familiarizing themselves with the assessment of image quality, which is probably the reason for the moderate degree of inter-observer agreement seen in this study.
- Inclusion of some patients whose body weight was ≥100 kg (which is associated with high image noise) made it difficult to apply the result of the present study.

## Conclusion

Investigation of the performance of DECTA in imaging peripheral arteries was carried out by comparing the efficacy of low volume of contrast medium with a routine volume of contrast medium. The outcome of the investigative study was that the image quality of DECTA obtained from both groups was clinically acceptable. The quality of DECTA peripheral artery images received at 65 keV and 50% ASIR, with low contrast medium volume protocol, was comparable to the images of the routine contrast medium volume DECTA images. It may therefore be concluded that lowering contrast medium volume by 50% could lead to diagnostically satisfying images during DECTA of peripheral arteries, at the same time reducing the risk of CIN in susceptible patients. Needless to say, more detailed and substantial research is necessary to reiterate the findings of the present investigation.

## Supporting Information

S1 Table Effects of dependent variables and scanning time during dual-energy CT angiography. (DOCX)

S2 Table Dependent variables consisting of age, hypertension, diabetes and body weight in relation to the scanning time. (DOCX)

S3 Table Estimated margin mean values between these variables and contrast groups. (DOCX)
