# Correction: Dual Energy CT Angiography of Peripheral Arterial Disease: Feasibility of Using Lower Contrast Medium Volume

**DOI:** 10.1371/journal.pone.0145976

**Published:** 2015-12-23

**Authors:** Abdulrahman Almutairi, Zhonghua Sun, Abduljaleel Poovathumkadavi, Tarek Assar

The image for Fig 2 is incorrect. Please see the corrected Fig 2 here.

**Fig 2:**
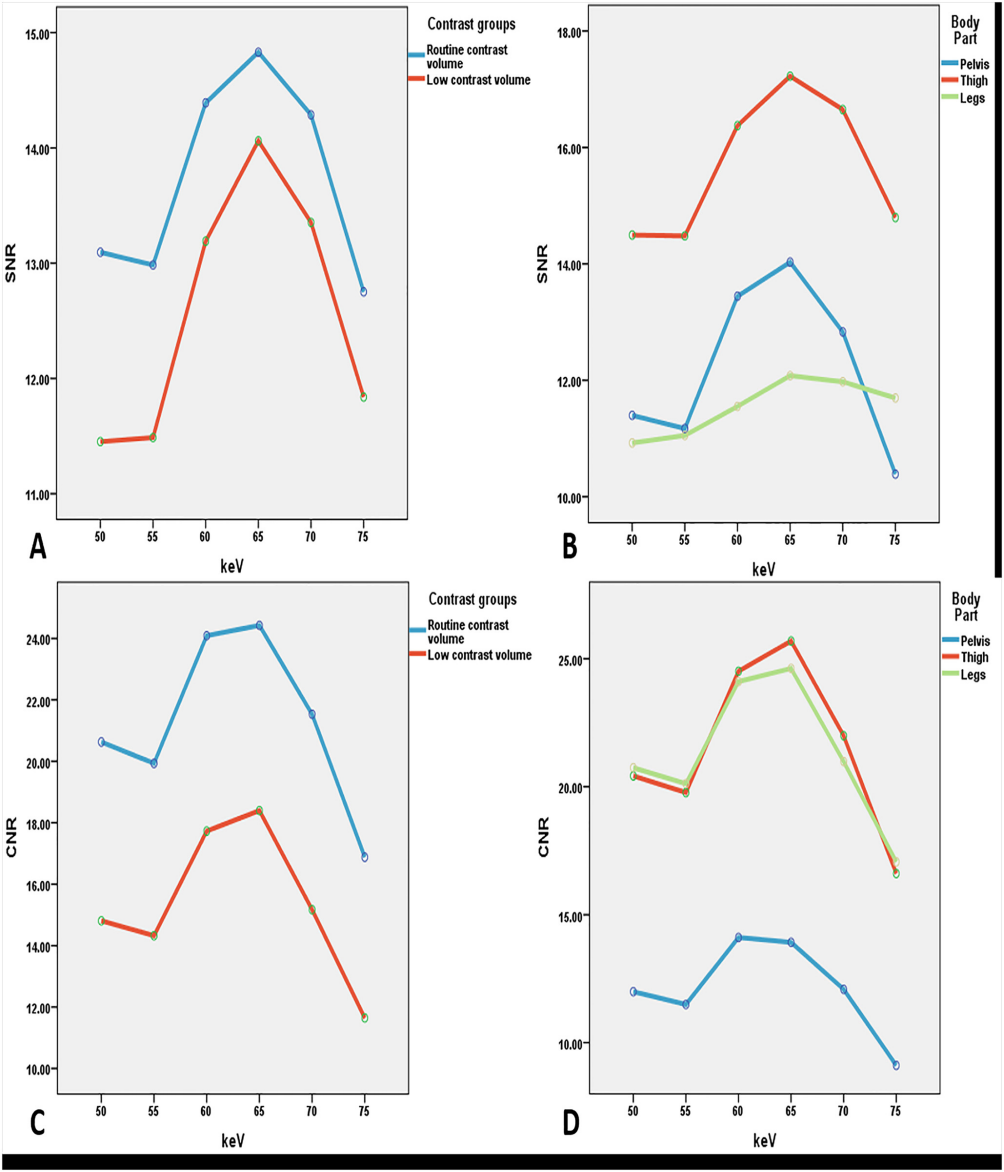
Comparison of SNR and CNR measured at two contrast groups at different body parts with variable keV sets. Comparison of calculated and measured SNR in monochromatic images with A showing the differences between the two contrast groups at different keV sets, B representing the SNR values of keV sets with three body parts, C showing the comparison of calculated and measured CNR in monochromatic images for the two contrast groups, and D demonstrating the CNR values of three body parts at different keV sets. In the range of 55–65 keV, both of the two curves increase sharply with the gradual rise in keV. Between 65 and 75 keV, both curves of the contrast values decrease sharply with 65 keV resulting in the highest value. SNR: signal-to-noise ratio, CNR: contrast-to-noise ratio.
