# Supplementary material for: Dual Energy CT Angiography of Peripheral Arterial Disease: Feasibility of Using Lower Contrast Medium Volume
Source: PLoS One. 2015 Sep 29;10(9):e0139275. doi: 10.1371/journal.pone.0139275 (PMC4587806; doi:10.1371/journal.pone.0139275)
Supplement: S1 Table — (DOCX) [file pone.0139275.s001.docx]

**S1 Table: Tests of Between-Subjects**

| Effects Dependent Variable: Scanning time | | | | | |
| --- | --- | --- | --- | --- | --- |
|  | | | | | |
| Source | Type III Sum of Squares | df | Mean Square | F | Sig. |
| Corrected Model | 37.699^a^ | 6 | 6.283 | 6.964 | .000 |
| Intercept | 354.550 | 1 | 354.550 | 392.937 | .000 |
| Group | 1.078 | 1 | 1.078 | 1.195 | .285 |
| Gender | 10.768 | 1 | 10.768 | 11.934 | .002 |
| Hypertension | 6.422 | 1 | 6.422 | 7.117 | .013 |
| Diabetes | 8.942 | 1 | 8.942 | 9.910 | .004 |
| Age | 4.669 | 1 | 4.669 | 5.175 | .032 |
| Weight | 15.138 | 1 | 15.138 | 16.777 | .000 |
| Error | 21.655 | 24 | .902 |  |  |
| Total | 18550.179 | 31 |  |  |  |
| Corrected Total | 59.355 | 30 |  |  |  |
| a. R Squared = .635 (Adjusted R Squared = .544) | | | | | |
