# Supplementary material for: Dual Energy CT Angiography of Peripheral Arterial Disease: Feasibility of Using Lower Contrast Medium Volume
Source: PLoS One. 2015 Sep 29;10(9):e0139275. doi: 10.1371/journal.pone.0139275 (PMC4587806; doi:10.1371/journal.pone.0139275)
Supplement: S2 Table — (DOCX) [file pone.0139275.s002.docx]

**S2 Table Parameter Estimates**

| Dependent Variable: Scanning time | | | | | | |
| --- | --- | --- | --- | --- | --- | --- |
| Parameter | B | Std. Error | t | Sig. | 95% Confidence Interval | |
|  |  |  |  |  | Lower Bound | Upper Bound |
| Intercept | 22.040 | 1.178 | 18.708 | .000 | 19.608 | 24.471 |
| [Group=1] | -.404 | .369 | -1.093 | .285 | -1.166 | .358 |
| [Group=2] | 0^a^ | . | . | . | . | . |
| [Gender=1] | 1.389 | .402 | 3.455 | .002 | .559 | 2.219 |
| [Gender=2] | 0^a^ | . | . | . | . | . |
| [Hypertension=1] | -1.302 | .488 | -2.668 | .013 | -2.309 | -.295 |
| [Hypertension=2] | 0^a^ | . | . | . | . | . |
| [Diabetes=1] | 1.499 | .476 | 3.148 | .004 | .516 | 2.481 |
| [Diabetes=2] | 0^a^ | . | . | . | . | . |
| Age | -.038 | .017 | -2.275 | .032 | -.072 | -.003 |
| Weight | .047 | .011 | 4.096 | .000 | .023 | .070 |

a. This parameter is set to zero because it is redundant.
