# Supplementary material for: Dual Energy CT Angiography of Peripheral Arterial Disease: Feasibility of Using Lower Contrast Medium Volume
Source: PLoS One. 2015 Sep 29;10(9):e0139275. doi: 10.1371/journal.pone.0139275 (PMC4587806; doi:10.1371/journal.pone.0139275)
Supplement: S3 Table — (DOCX) [file pone.0139275.s003.docx]

**S3 Table Estimated Marginal Means**

| **1. Grand Mean** | | | |
| --- | --- | --- | --- |
| Dependent Variable: Scanning time | | | |
| Mean | Std. Error | 95% Confidence Interval | |
|  |  | Lower Bound | Upper Bound |
| 24.119^a^ | .203 | 23.701 | 24.538 |

a. Covariates appearing in the model are evaluated at the following values: Participant age = 52.84, Weight = 74.5484.

| 2. Contrast groups | | | | |
| --- | --- | --- | --- | --- |
| Dependent Variable: Scanning time | | | | |
| Contrast groups | Mean | Std. Error | 95% Confidence Interval | |
|  |  |  | Lower Bound | Upper Bound |
| 1 Routine contrast volume | 23.917^a^ | .280 | 23.339 | 24.496 |
| 2 Low contrast volume | 24.321^a^ | .268 | 23.768 | 24.874 |
|  | | | | |

a. Covariates appearing in the model are evaluated at the following values: Participant age = 52.84, Weight = 74.5484.

| 3. Gender | | | | |
| --- | --- | --- | --- | --- |
| Dependent Variable: Scanning time | | | | |
| Gender | Mean | Std. Error | 95% Confidence Interval | |
|  |  |  | Lower Bound | Upper Bound |
| 1 Male | 24.814^a^ | .233 | 24.332 | 25.295 |
| 2 Female | 23.425^a^ | .330 | 22.745 | 24.105 |
|  | | | | |

a. Covariates appearing in the model are evaluated at the following values: Participant age = 52.84, Weight = 74.5484.

| 4. Hypertension | | | | |
| --- | --- | --- | --- | --- |
| Dependent Variable: Scanning time | | | | |
| Hypertension | Mean | Std. Error | 95% Confidence Interval | |
|  |  |  | Lower Bound | Upper Bound |
| 1 Yes | 23.468^a^ | .274 | 22.903 | 24.034 |
| 2 No | 24.770^a^ | .355 | 24.037 | 25.503 |
|  | | | | |

a. Covariates appearing in the model are evaluated at the following values: Participant age = 52.84, Weight = 74.5484.

| 5. Diabetes | | | | |
| --- | --- | --- | --- | --- |
| Dependent Variable: Scanning time | | | | |
| Diabetes | Mean | Std. Error | 95% Confidence Interval | |
|  |  |  | Lower Bound | Upper Bound |
| 1 Yes | 24.869^a^ | .305 | 24.238 | 25.499 |
| 2 No | 23.370^a^ | .320 | 22.710 | 24.030 |
|  | | | | |

a. Covariates appearing in the model are evaluated at the following values: Participant age = 52.84, Weight = 74.5484.
